# Assessment of mathematical model for elliptical excision: solving the doubt about vertex angle and predicting postoperative wound length

**DOI:** 10.1186/s12893-023-02234-w

**Published:** 2023-10-27

**Authors:** Rifeng Zou, Feng Lin, Chunyu Hao, Dapeng Zhou, Jiulong Liang, Hongyi Wang

**Affiliations:** Department of Burn and Plastic, General Hospital of Northern Theater Command, No.83, Wenhua Road, Shenhe District, Shenyang, Liaoning Province 110016 China

**Keywords:** Elliptical excision, Mathematical model, Final wound length

## Abstract

**Background:**

Elliptical excision is the most commonly used method for small benign tumour excision and primary closure. However, elliptical excision remains the topic of debate. The aim of this study was to explore the relationship among postoperative incision, vertex angle, and the length and width of fusiform excision through a mathematical model.

**Methods:**

We collected data from fusiform circle excisions performed at the author’s hospital (101 cases). The measured values were applied to the mathematical model formula for statistical analysis.

**Results:**

The functional relationships among the length, width, arc, and angle of the fusiform circle were obtained. The mean apical tangent angle was 100.731°±15.782°, and the mean apical inner angle was 50.366°±7.891°. There was no significant difference between the preoperatively designed arc length preoperative and the postoperative incision length (P < 0.001). The apical vertex push-out distance equals half of the value of the fusiform length subtracted from arc.

**Conclusions:**

The mathematical model can be used to design the incision for ellipse fusiform excision to predict the final wound length.

## Introduction

The most commonly used method for benign tumours excision or primary closure is elliptical or fusiform excision [1–5]. In the past, “elliptical” excision was used more frequently [6]. Notably, literal interpretation of the term “elliptical” is not applicable [6]. The terms “elliptical” and “fusiform” resection are used interchangeably in the literature, both used to describe the same shape [7–9]. The term “fusiform” may be more accurate [10], but fusiform is not a strictly defined shape. All shapes, wide in the middle and tapered at both ends, can be called fusiform [7]. Some surgeons believe that a fusiform ellipse is the most common fusiform excision pattern, which is formed by the overlapping area of two ellipses [11]. However, some surgeons believe that a classic fusiform is formed by the arcs of circles [10]. Although an arc is the most familiar curve for most persons. It is difficult for a person without professional training to draw a target curve (especially ellipses) accurately. Therefore, it seems more feasible for surgeons to draw a fusiform circle when designing an incision.

In designing a fusiform incision, the designer would focus on the long axis and the vertex angle to avoid causing a dog ear deformity and to control the appearance of the postoperative scar [12, 13]. Most surgeons believe that the apical angle is less than 30 degrees and the length-width ratio is 3:1 [7, 8, 12, 14]. Mathematical models of two fusiform shapes have been established and discussed in terms of mathematical deduction, such as the length-width ratio, vertex angle, and the length of the postoperative scar. When the length-to-width ratio is 3:1, the vertex angle is 74°, and the radius of the circular arc is 5 times the short axis of the fusiform [15]. These mathematical models described in articles are basically consistent and have their points of emphasis. The description of the relationship between the vertex angle and the length-to-width ratio is consistent. However, the models have yet to be comprehensively elucidated, and all mathematical models have not been cerified by clinical data thus far.

Previous studies have noted that elliptical excision remains a heavily debated topic. In general, we believe that mathematical models can improve the understanding of “elliptical” and “fusiform” excision, especially in terms of the apical angle. To create a mathematical model, an accurate preoperative incision length and an accurate postoperative incision length can be calculated. In this study, an equal diameter intersecting circle model of the fusiform circle will be established, and we will comprehensively and systematically describe its mathematical principle. In addition, accurate clinical resection data will be collected and corrected to validate and evaluate the mathematical model.

## Methods

### Equal diameter intersecting circle model of the fusiform circle

**Fig. 1:**
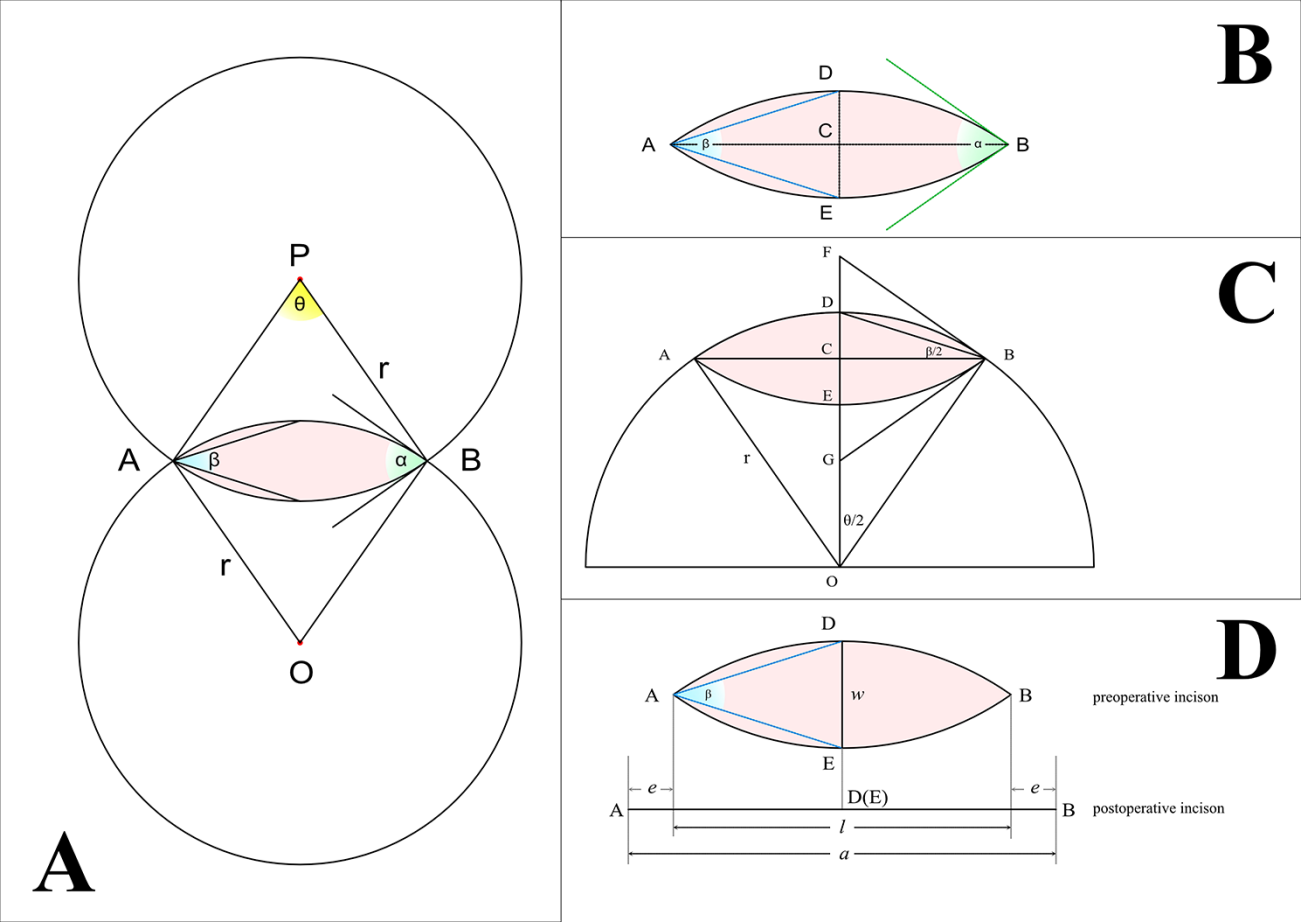
Equal diameter intersecting circle model of the fusiform circle; **A**: two identical circles (centre O and P, radius is r) intersected at points A and B. The intersected area is the fusiform shape, which is the target excised section. The arcs (𝑎) between points A and B derived from the two identical circles are identical. With centre O as the vertex, the angle formed by points A, O and B is the central angle (𝜃). With point A or B as the vertex, the angle formed by tangents of circles at point A or B is named as the apical tangent angle (𝛼). **B**: The straight-line connecting points A and B represents the fusiform length (𝑙). The line connecting the centres of the two circles intersects the fusiform major axis (AB) at point C and separately intersects the identical arcs at points D and E. The straight line between points D and E is the fusiform width (𝑤). With the endpoint of the fusiform as the vertex (point A or B), the angle formed by the endpoints of the fusiform minor axis (DE) is named the apical inner angle (𝛽). **C**: BF and BG are the tangent lines at point B, ∠AOB=𝜃, ∠FBG=𝛼, ∠DAE= 𝛽. **D**: Preoperative incision design and final wound after suture

As shown in Fig. 1, two identical circles (centre O and P, radius is r) intersect at points A and B. The intersected area is the fusiform shape, which is the target excision section, with point A and point B as the endpoints of the fusiform shape. The arcs (𝑎) between points A and B derived from the two identical circles are identical. The straight-line connecting points A and B represents the fusiform length (𝑙). The line connecting the centres of the two circles intersects the fusiform major axis (AB) at point C and separately intersects the identical arcs at points D and E. The straight line between points D and E is the fusiform width (𝑤). With centre O as the vertex, the angle formed by points A, O and B is the central angle (𝜃). With point A or B as the vertex, the angle formed by tangents of circles at point A or B is named the apical tangent angle (𝛼). With the endpoint of the fusiform as the vertex (point A or B), the angle formed by the endpoints of the fusiform minor axis (DE) is named the apical inner angle (𝛽). As it shown in Fig. 1, ∠AOB (the central angle) =𝜃, ∠FBG (the apical tangent angle) =𝛼, ∠DAE (the apical inner angle) =𝛽, AB (fusiform length) =𝑙, DE (fusiform width) =𝑤.

The central angle equals the apical tangent angle, which is twice the apical inner angle, and it can be proven by the Pythagorean theorem and the theorem of angle bisector (Formula 1).

Formula 1 $$\theta = \alpha = 2\beta$$

Formula 2 $${\beta }=2{\text{tan}}^{-1}\frac{w}{l}$$

The functional relationships among 𝛽, 𝑤, and 𝑙 can be established through tangent function (Formula 2). The functional relationship between 𝑙, 𝑎 and 𝛽 can also be derived (Formula 3 and Formula 4).

Formula 3 $$l=\left(cot\frac{{\beta }}{2}\right)w$$

Formula 4 $$\eqalign{ arc & =\frac{{\theta }}{360^\circ }\pi r \cr & =\frac{{\beta }}{360^\circ }\pi \left(1+{\left(\text{cot}\frac{{\beta }}{2}\right)}^{2}\right)w }$$

## Clinical data collection

### Operation data

**Fig. 2:**
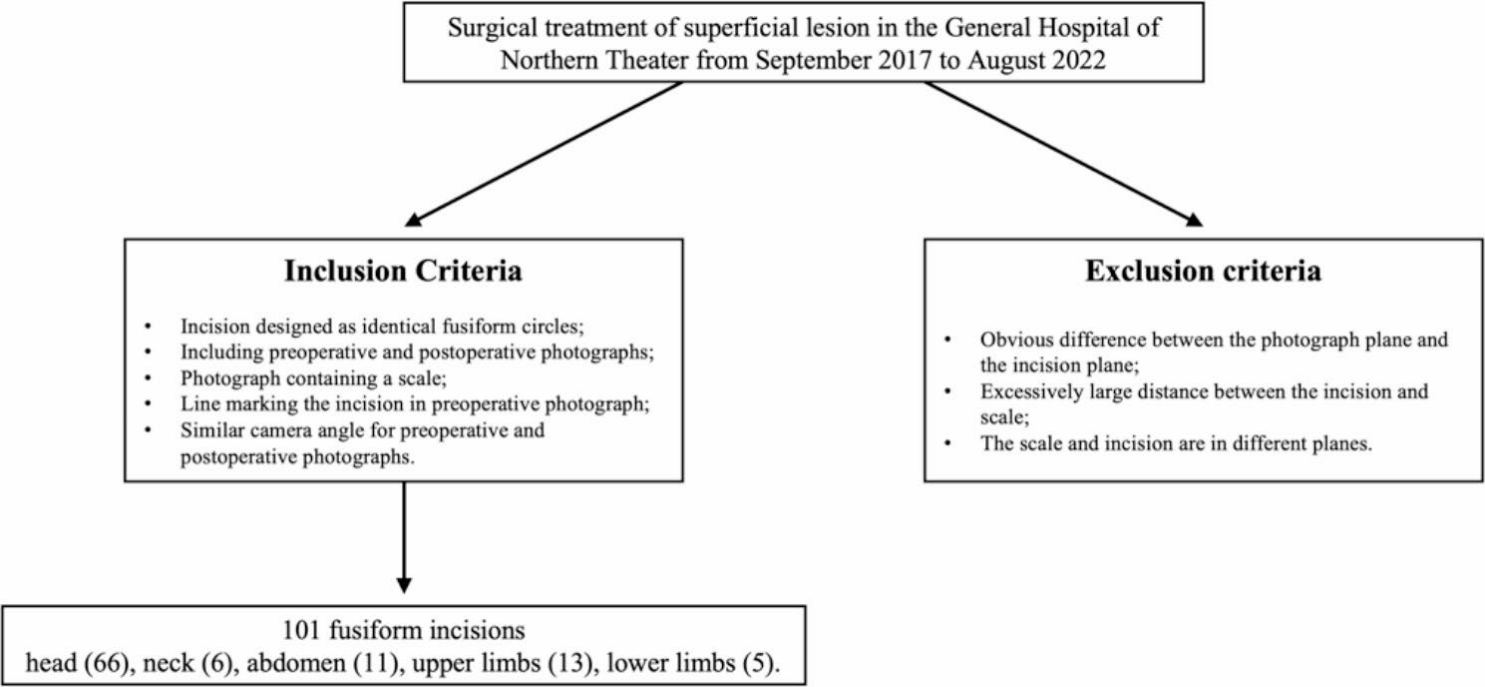
Flow diagram of the selection of the studied population

Photographs of the surgical treatment of superficial lesion in the General Hospital of Northern Theater taken from September 2017 to August 2022 were selected. The inclusion criteria were as follows: (1) incision designed as identical fusiform circles; (2) including preoperative and postoperative photographs; (3) photograph containing a scale; (4) line marking the incision in preoperative photograph; and (5) similar camera angle for preoperative and postoperative photographs. Exclusion criteria: (1) obvious difference between the photograph plane and the incision plane; (2) excessively large distance between the incision and scale; and (3) the scale and incision are in different planes (Fig. 2).

Finally, we included 101 patients with 101 fusiform incisions in the head (66), neck (6), abdomen (11), upper limbs (13), and lower limbs (5) (Table 1).

**Table 1:** Demographic and Location of the Study Population

Overall(N = 101)		
Mean Age (SD)		37.0(14.5)
Gender	Female	58 (57.4%)
	Male	43 (42.6%)
Location	Head	66 (65.3%)
	Neck	6 (5.9%)
	Abdomen	11 (10.9%)
	Upper limbs	13 (12.9%)
	Lower limbs	5 (5.0%)

### Measuring method

**Fig. 3:**
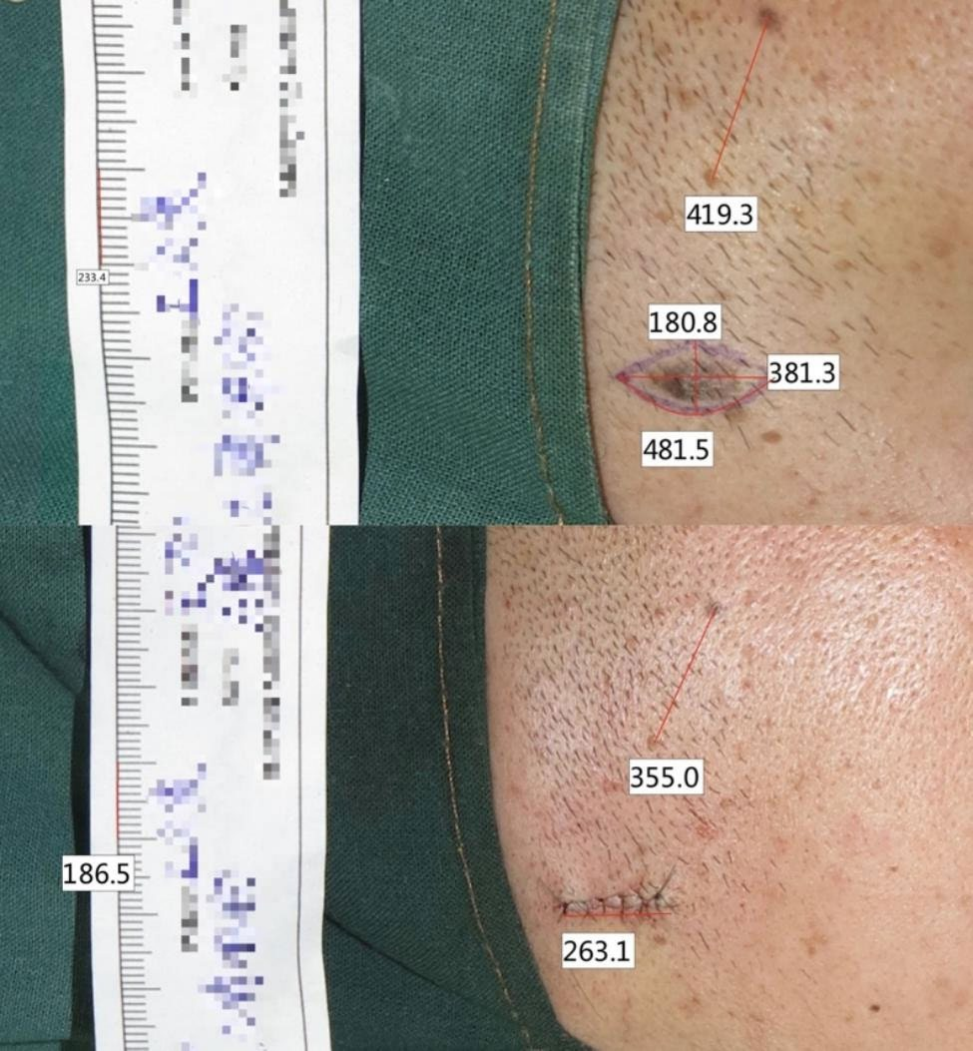
Corrected preoperative and postoperative photograph values by scale and reference object to obtain relative values for comparison

Measurements on photographs were calculated by Camera Measure software (2.1.3.250). Measurements include length, width, one-sided designed preoperative incision (pre-i), preoperative scale (pre-s), preoperative reference (pre-r), postoperative incision (post-i), postoperative scale (post-s), postoperative reference (post-r). (Fig. 3)

Altitude discrepancies are corrected by the scale and reference object for preoperative and postoperative photographs (Fig. 3). The corrected preoperative length = measured length/preoperative scale. The corrected postoperative length = (measured postoperative length * preoperative reference)/(postoperative scale * postoperative reference).

### Statistical analysis

Statistical analysis was performed by R version 4.2.3. We obtained arc (𝑎), central angle(𝜃), apical tangent angle (𝛼), and apical inner angle (𝛽) by model calculation with the data of fusiform length (𝑙) and postoperative width (𝑤), and compared them with arc AB (𝑎) and the designed preoperative incision (pre-i) by t-test (P < 0.05).

## Results

**Fig. 4:**
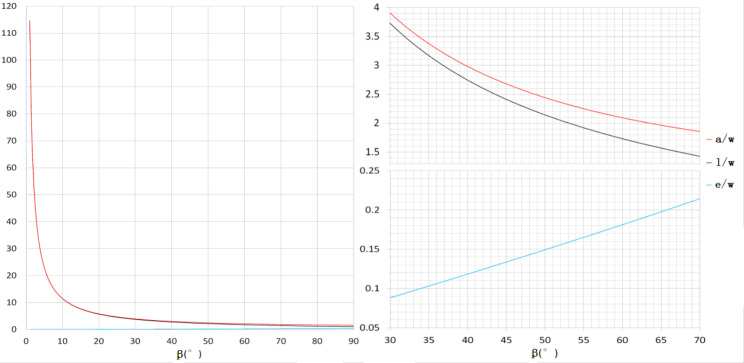
Arc-width ratio (𝑎/𝑤); length-width ratio (𝑙/𝑤); ratio of vertex pushed-out distance with width (e/w), and the relationship with apical inner angle (𝛽)

1. We obtained the functional relationship among fusiform length (𝑙), fusiform width (𝑤), arc (𝑎), apical tangent angle (𝛼), and apical inner angle(𝛽).
2. According to the model, the mean arc length was 1.515 ± 0.593 cm, the mean apical tangent angle was 100.731°±15.782°, and the mean apical inner angle was 50.366°±7.891° (Table 2). Therefore, we further evaluated whether the model had the ability to simulate the pattern of the fusiform incision and to predict whether the postoperative incision would be the same.
3. After data correction, the mean preoperative width was 0.612 ± 0.215 cm, the mean preoperative length was 1.330 ± 0.550 cm, the mean preoperatively designed arc was 1.511 ± 0.599 cm, and the mean postoperative incision was 1.555 ± 0.735 cm (Table 2). There was a significant difference between the preoperatively designed length and the postoperative incision length (P < 0.001), with a coefficient of correlation of 0.917 (P < 0.001) (Table 3). There was no significant difference between the preoperatively designed arc length and the postoperative incision length (P = 0.64) (Table 4). Therefore, there is a strong correlation between the postoperative incision made in fusiform excision and the fusiform length (𝑙). However, they are not equal; instead, the postoperative incision length depends on the arc of the fusiform.
4. According to results above, the preoperatively designed fusiform arc length equals the postoperative incision length. The pushed-out distance of the preoperatively designed fusiform apical vertices equals half of the value of the fusiform length (𝑙) subtracted from the arc (𝑎). With Formula 3 and Formula 4, we finally obtain Formula 5, the function between 𝑎/𝑤 and 𝛽 (Fig. 4).

Formula 5 $$\eqalign{\text{a} & =\frac{1}{2} \left[\frac{{\beta }}{360^\circ }\pi \left(1+ {\left(\text{cot}\frac{{\beta }}{2}\right)}^{2}\right) \right. \cr &\left. -\left(cot\frac{{\beta }}{2}\right)\right]w }$$

**Table 2:** Descriptive of clinical and mathematical model values

	Head (N = 66)	Neck (N = 6)	Abdomen (N = 11)	Upper Limbs (N = 13)	Lower Limbs (N = 5)	Overall (N = 101)
Preoperative Width (cm) Mean (SD)	0.565(0.175)	0.650(0.182)	0.726(0.290)	0.702(0.232)	0.702(0.361)	0.612(0.215
Preoperative Length (cm) Mean (SD)	1.23(0.428)	1.25(0.254)	1.57(0.770)	1.50(0.638)	1.80(1.05)	1.33(0.550)
Preoperative Designed Arc (cm) Mean (SD)	1.39(0.450)	1.50(0.441)	1.82(0.860)	1.70(0.701)	1.95(1.12)	1.51(0.599)
Model Arc (cm) Mean (SD)	1.40(0.462)	1.46(0324)	1.79(0.827)	1.72(0.680)	1.97(1.13)	1.51(0.594)
Postoperative Incision (cm) Mean (SD)	1.38(0.525)	1.51(0.325)	1.94(0.912)	1.92(1.11)	2.15(1.16)	1.55(0.735)
Apical Inner Angle (degree) Mean (SD)	100(16.3)	109(9.57)	101(15.6)	104(15.7)	89.1(12.0)	101(15.8)
Apical Tangent Angle/Central Angle (degree) Mean (SD)	50.1(8.16)	54.3(4.79)	50.5(7.78)	51.9(7.85)	44.5(5.98)	50.4(7.89)

**Table 3:** Comparison of preoperative design length and postoperative incision length

Groups	Head(66)	Neck(6)	Abdomen(11)	Upper Limb(13)	Lower Limb(5)	Overall(101)
Preoperative Design Length	1.39(0.450)	1.50(0.441)	1.82(0.860)	1.70(0.701)	1.95(1.12)	1.511(0.599)
Postoperative Incision Length	1.38(0.525)	1.51(0.325)	1.94(0.912)	1.92(1.11)	2.15(1.16)	1.555(0.735)
*r*						0.917
*P*						< 0.001

**Table 4:** Comparison of preoperative design arc length and postoperative incision length

Goups	Head(66)	Neck(6)	Abdomen(11)	Upper Limb(13)	Lower Limb(5)	Overall(101)
Preoperative Designed Arc Length	1.23(0.428)	1.25(0.254)	1.57(0.770)	1.50(0.638)	1.80(1.05)	1.33(0.550)
Postoperative Incision Length	1.38(0.525)	1.51(0.325)	1.94(0.912)	1.92(1.11)	2.15(1.16)	1.555(0.735)
*P*						0.64

## Discussion

In our circle model, the apical divides into the apical tangent angle and the apical inner angle. The apical inner angle equals half of the apical tangent angle in the equal diameter intersecting circle model. The apical tangent angle is the angle that is usually referred to as the apical angle or vertex angle in the current literature. The circle model we built is similar to Moody’s model and Rocher’s model. Depending on clinical case data, the mean length-width ratio was 2.18 ± 0.39, and the mean apical tangent angle was 100.73 ± 15.78 degrees. The results are almost consistent with the results reported in the published literature. According to the custom elliptical or fusiform design procedure, the length-width ratio (diameter of circular lesion) is 3 ~ 4:1, and the apical angle less is than 30 degrees [7, 9, 12, 14]. Tilleman et al., through a mathematical model, found that when the length-width ratio is 3, the apical angle is 44 ~ 74 degrees, and when the ratio equals 4:1, the apical angle is 33 ~ 56 degrees [11]. The apical angle reached 30 degrees only under the rhombus or S pattern with a length-width ratio of 3 ~ 4 [11]. Moody et al. used a circle model to simulate that the length-width ratio is 3:1, the apical angle is 74 degrees, and the ratio reaches 7.6:1 when the apical angle is 30 degrees [15]. In addition, Tilleman et al. combined published literature reports with their own cases data. When the ratio is 3 ~ 4, the apical angle is 58 ~ 48 degrees in the published literature report and 59 ~ 47 degrees in their case data [16]. There were significant differences among published literature data, clinical data, and theoretical data because of measurement errors in clinical practice [16, 17]. The apical angle is an important part of fusiform design. According to the theoretical model, published literature and clinical case data, the apical angle or vertex angle range is significantly more than 30 degrees. This situation may result in different design procedures, and the apical angle may not be the same angle. If the vertex angle or apical angle is less than 30 degrees, we recommend defining the apical angle as the apical inner angle rather than as the apical tangent angle. This result is quite different from our intrinsic conceptions, this problem should be prioritized and receive more attention.

The apical tangent angle is formed by the fusiform pattern vertex and width vertices. Tilleman et al. compared ellipse, circle, rhomboid, mosque and S shape mathematical models, and there were no significant differences [11]. It looks like the angle of a rhombus shape, but they are totally different. The angle of a true rhombus pattern excision is larger than the apical inner angle in the circle fusiform. With the same ratio, the apical angle formed by the lesion margin and vertex point in the lemon (American Football) shape or rhombus shape is quite different from the sharp apical angle of a circular fusiform [18]. The apical inner angle is determined by the length width ratio of the fusiform, and the fusiform can be precisely measured and drawn accurately. This may also be one of the reasons for the difference between traditional cognition of vertex angle and the current research conclusions.

There was a significant difference between the preoperatively designed length and the postoperative incision length. The postoperative incision length depends on the arc of the fusiform rather than the fusiform length [19, 20]. This is another unnoticed detail about fusiform excision before. A circle fusiform design is the most commonly used pattern and is the presupposition for model building. One of the reasons is that circles are the most familiar shapes or curves, and arcs should be closer to circles in the human being subconscious [15, 21]. In our study, the measured values are not significantly different from the theoretical model values. It is easier to predict with circles than ellipses and can be used for final wound length prediction. To ensure the design fusiform, what we want is the circle fusiform, the procedure of preoperative draw excision line should be completed in detail.

Most surgeons believe that a large apical angle results in dog-ear formation [14]. Mathematical model studies have provided evidence to rectify this cognition. Surgeons attempt to shorten the wound length or reduce soft tissue waste and avoid postoperative dog ears by innovative techniques [10, 13, 22, 23]. Ishihara et al. avoided postoperative dog ears by cutting off the subcutaneous fibrous tissue and shortening the final wound length [24]. Tilleman et al. only excised out the lesion along edges and achieved prevention dog ears through wide subcutaneous dissociation and special suture techniques [25]. These studies challenge the conventional view on how to prevent postoperative dog ear. The length width ratio and apical angle may not be the major factors. Skin elasticity and the capacity for rearrangement are other factors [26–29]. When the rearrangement of local tissue is out of its capacity, dog-ear appears. A modified processor, as described in the literature, aiming at increasing soft tissue capacity, can reduce the probability of dog-ear. Soft tissue displacement is mostly concentrated at vertex point of fusiform excision, where it just forms dog ears. Additionally, a function of postoperative vertex length was found in our model, which is related to the apical inner angle. If the diameter of the lesion is large, lengthening the surgical incision and reducing apical inner angle are controlling factors for postoperative displacement. This conclusion is consistent with clinical experience. It is also possible to determine the shortest incision length with the most displacement of soft tissue location to solve the dog-ear problem with reverse thinking and further clinical data research. The relationship between the apical tangent angle and dog-ear formation needs to be confirmed by further research.

In this study, the sample size was small. For the mathematical model to be more reliable, more clinical data are needed to verify its validity. The measured data are from different individuals, ages, sex, and body areas. Skin elasticity and redistribution capacity are different in each body are and may be even greater in different individuals. This may be a potential factor influencing the final result. During the clinical data collection, we used software to measure the parameters on the photograph and corrected the preoperative and postoperative photograph values by scale and reference object to obtain relative values for comparison. The measurement error is not neglected, and we correct errors through the ratio relationship of reference objects by stereo rectification [30]. The accuracy of clinical data values is better than that measured directly on the lesion, which significantly improved the reliability of model prediction. Most of the lesions in clinical cases are 0.24–1.31 cm wide and 0.62–3.55 cm long. At this size, sample excision is better than local flap. For lesions with a larger size in another area, the postoperative incision may be more susceptible to soft tissue displacement, and the model for this situation needs more data to validated. The model used in our study was built on sample equal diameter intersecting circles. In this case, the centre angle equals twice the apical tangent angle. There is an obvious functional relationship between circle fusiform length and width. This is the characteristic of the intersecting circle model, showing the beauty of mathematics and geometry, and has theoretical value.

## Conclusions

In summary, through mathematical model derivation and clinical data verification, the relationship of the centre angle, apical tangent angle and apical inner angle can be used to form a standard method of fusiform excision design to advance the basic understanding of surgery. The postoperative incision length depends on the arc of the fusiform rather than the fusiform length, and the mathematical model also provides an effective model for final wound length prediction.

## Data Availability

The datasets generated and analyzed during the current study are not publicly available but are available from the corresponding author on reasonable request. If someone wants to request the data from this study, please contact Wang Hongyi M.D, Email: whyi87@163.com.
